# Aflatoxin B_1_ (AFB_1_) biodegradation by a lignolytic phenoloxidase of *Trametes hirsuta*

**DOI:** 10.1038/s41598-025-90711-y

**Published:** 2025-02-21

**Authors:** Tuncay Söylemez, Ralf Günter Berger, Ulrich Krings, Mustafa Yamaç

**Affiliations:** 1 Institut für Lebensmittelchemie, Gottfried Wilhelm Leibniz Universität Hannover, Callinstraβe 5, 30167 Hannover, Germany; 2 Faculty of Science and Letters, Department of Biology, Eskisehir Osmangazi University, Eskisehir, Turkey

**Keywords:** Aflatoxin B_1_, Biodegradation, Enzyme purification, Phenoloxidase, *Trametes hirsuta*, Biotechnology, Microbiology

## Abstract

Aflatoxin B_1_ (AFB_1_) is a highly potent mycotoxin that poses a serious threat to human and animal health. This study investigated the biodegradation of AFB_1_ by the supernatant of submerged cultured *Trametes hirsuta*, with a focus on identifying and characterizing the responsible enzyme(s). The extracellular enzymes of the white-rot mushroom were extracted from the supernatant and pre-separated using anion exchange fast protein liquid chromatography (FPLC). To pinpoint the specific enzyme, the eluted protein fractions exhibiting the highest degradation activity were subjected to detailed biochemical and proteomic analyses. A second purification step, ultrafiltration, yielded an electrophoretically pure enzyme. Sequencing of tryptic peptides using a nano-LC system coupled to a qQTOF mass spectrometer identified the enzyme as a lignolytic phenoloxidase. The enzyme exhibited a molecular mass of 55.6 kDa and achieved an impressive AFB_1_ degradation rate of 77.9% under optimized experimental conditions. This is the first fungal lignolytic phenoloxidase capable of aflatoxin degradation without requiring hydrogen peroxide as a cofactor, highlighting its unique catalytic mechanism. It may be used in mycotoxin remediation strategies, such as treating the surfaces of contaminated fruits, vegetables, and nuts.

## Introduction

Mycotoxins are hazardous fungal metabolites, which contaminate crops and food products, especially when they are exposed to high temperatures and moisture during the pre- and post-harvest stages. The most harmful mycotoxins are aflatoxins, which are mostly produced by *Aspergillus flavus* and *Aspergillus parasiticus* strains. Because of their carcinogenic potential, aflatoxins pose a serious risk to both human and animal health. The most potent aflatoxin, B_1_ (AFB_1_), is a strong hepatocarcinogen with mutagenic and teratogenic properties. Thus, preventative steps to guarantee the safety of food and feed have to be taken^1–3^.

**Fig. 1:**
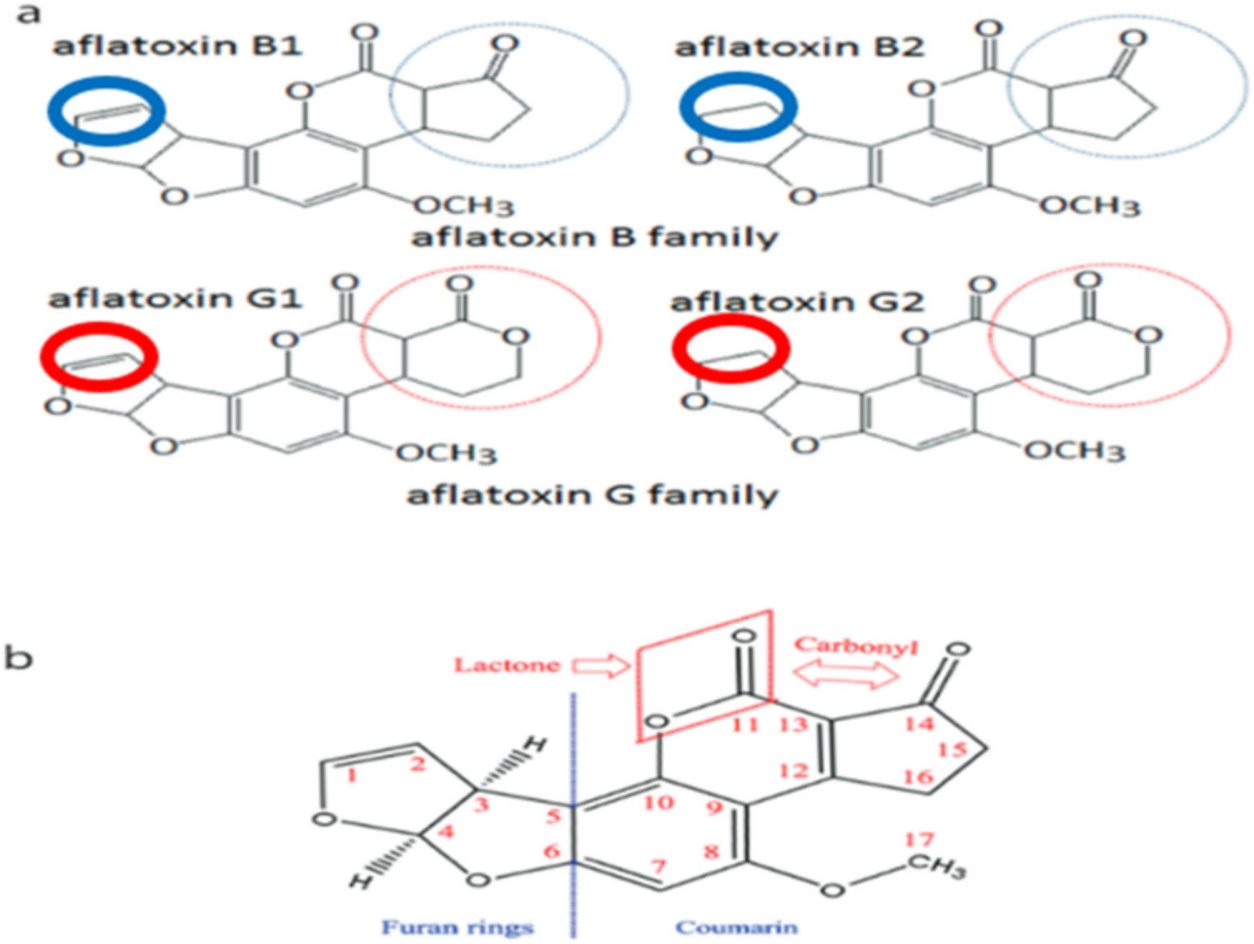
(**a**) Chemical structure of Aflatoxins of B family (B_1_ and B_2_) and G family (G_1_ and G_2_). The moiety in blue or red Circle shows the difference between the two groups, and (**b**) Chemical structure of major functional moieties of Aflatoxins^4^.

AFB_1_, with a molecular formula of C_17_H_12_O_6_ and a molecular mass of 312.27 g/mol, is a white, odourless, and heat-resistant crystalline solid and is considered the most toxic member of the aflatoxin group^4,5^. AFB_1_ has a complex chemical structure containing four fused rings. (Fig. 1). In human metabolism AFB_1_ is biotransformed oxidatively to the toxic product AFB_1_-8,9-epoxide^6^. The two electrophilic carbon atoms at the epoxide ring are highly reactive and this leads to the formation of covalent bonds with DNA, RNA, and proteins, causing adverse effects such as DNA damage, mutations, protein synthesis inhibition, and enzyme deactivation. Due to its high chemical stability, AFB_1_ can remain in food and feed for a long time, posing a significant risk to food safety.

AFB_1_ often contaminates food products such as peanuts, maize, and tree nuts and consumption may causes liver damage, immune system suppression and cancer. Affecting approximately 25% of the global food supply, aflatoxin contamination places a particularly heavy burden on developing countries. Up to 40% of food crops in sub-Saharan Africa are potentially contaminated. In addition to direct health impacts, aflatoxin contamination results in increased food waste and reduced crop yields and trade. Mitigation efforts include improved agricultural practices, resistant crop varieties, and regulatory measures such as maximum allowable limits. However, despite these measures, aflatoxin contamination remains a significant global public health issue, highlighting the need for ongoing research to develop effective strategies to reduce its impact on global food safety^7–10^. According to the official journal of the European Commission, there are specific maximum levels for aflatoxin B_1_ in different food groups, ranging from 2 to 12 parts per billion (ppb) or micrograms per kilogram (µg/kg) (EC, 2016; https://eur-lex.europa.eu/legal-content/EN/TXT/PDF/?uri=CELEX:32006R1881)^11^.

Various solutions to reduce undesired contamination in drinking water, foods, and environmental samples has been investigated, amongst them photocatalysis or the use of nanocomposites, whose active principle is based on adsorption and/or complexation. Similarly, hazardous fungal metabolites like mycotoxins require effective degradation methods to mitigate their impact on food safety and public health^12,13^. Plant breeding programs and genetic approaches have been employed to develop resistance to AFB_1_-producing fungi. However, these methods face challenges such as consumers` concerns about genetically modified crops. These limitations further underscore the need for alternative approaches, such as enzymatic detoxification strategies, which are the focus of this study^14,15^.

Aflatoxins (AFs) are currently addressed by chemical and biological means for degradation or elimination. Chemical methods using strong oxidants such as chlorine dioxide and sodium hypochlorite can efficiently eliminate AFB_1_, but present disadvantages, such as high costs and the persistence of reagent residues, potentially causing secondary contamination. Physical methods, including visual inspection and gamma radiation, aim to reduce AFB_1_ levels, but each method has its limitations. Biological detoxification, involving microorganisms and their by-products, stands out for its specificity and effectiveness. Various approaches used genetically engineered plants as well as various microorganisms, such as bacteria, yeast, moulds, and macrofungi to screen for effective enzymes. Beside the limited chemical and physical approaches, these biological methods have emerged as promising, environmentally friendly, and sustainable alternatives for AFB_1_ degradation^16–18^.

Microorganisms, particularly *Bacillus* and *Pseudomonas* species, have demonstrated enzymatic activity that can detoxify AFB_1_ using various enzymatic pathways. For example, specific enzymes such as laccases and peroxidases have been identified in these microbes that contribute to the detoxification process. Some fungal species also convert AFB_1_ to non-toxic derivatives via enzymatic degradation pathways. Among the enzymes identified were aflatoxin-detoxifizyme (ADTZ) and a laccase from *Armillariella tabescens*, respectively^19–21^.

Various intracellular and extracellular enzymes from different organisms are involved in the degradation of AFB_1_. These include intracellular aflatoxin oxidase (AFO/ADTZ) from the higher fungus *Armillariella tabescens*, extracellular laccases from white rot fungi, peroxidase from *Pseudomonas* sp., reductase from *Mycobacterium smegmatis*, manganese peroxidase from *Pleurotus ostreatus*, and an AF degradation enzyme from *Myxococcus fulvus*^22^. As a result, many researchers have turned their attention to white rot fungi, investigating the purification of extracellular enzymes with stable lignolytic activity and evaluating their mycotoxin degradation potential^23–26^.

This study aimed to identify and characterize the enzyme(s) responsible for AFB_1_ degradation in supernatants of *Trametes hirsuta*, which was identified in our previous screening studies for AFB_1_ degradation^27^. *T. hirsuta* demonstrated remarkable degradation potential compared to other fungi. For the first time, this study describes the identification and biochemical characterization of a lignolytic phenoloxidase from *T. hirsuta* capable of cofactor free degrading AFB_1_.

The novelty of this research is further underscored by a PubMed analysis over the last five years, which reveals only 95 studies on enzymatic solutions for AFB_1_ degradation. Of these, only two addressed *Trametes* species, and none involved *T. hirsuta*, highlighting the unique contributions of this study. Importantly, lignolytic phenoloxidases are rare enzymes, with only one PubMed entry in the last decade^28^.

By addressing the limitations of existing chemical and biological methods, this work offers an environmentally friendly, scalable, and efficient solution to mycotoxin detoxification. These findings have significant implications for global food safety and could pave the way for further research into sustainable enzymatic detoxification strategies.

## Materials and methods

### Chemicals and reagents

All chemicals and reagents used in this study were of analytical grade. Aflatoxin B_1_ standard (purity ≥ 98%) was purchased from Sigma-Aldrich (Taufkirchen, Germany). All chemicals and medium components were obtained from Merck Millipore (Darmstadt, Germany).

### Biodegradation agent

The fungal isolate under investigation (coded as OBCC 5014) was isolated from a mushroom sample collected in Osmaneli, Bilecik, Türkiye. This particular isolate was selected among 94 macrofungal isolates for its AFB_1_ and OTA degradation capacity^27^. The isolate was preserved in the culture collection of the Leibniz University Hannover’s Institute of Food Chemistry under code # 329. By analysing the typical physical characteristics, visible both with the naked eye and under the microscope and comparing them with descriptions found in the relevant literature, the fungal sample was tentatively identified as a strain of *Trametes hirsuta*^29^. To confirm the traditional identification, the sequence of the internal transcribed spacer (ITS) rDNA region of the isolate was determined and then compared to sequences available in the GenBank database (http://www.ncbi.nlm.nih.gov/genbank/). For that purpose, the isolate 329 was grown on standard nutrient liquid - Agar (SNL) medium (g/L; D (+)-glucose monohydrate 30.0, L-asparagine monohydrate 4.5, potassium dihydrogen phosphate 1.5, magnesium sulphate hydrate 0.5, yeast extract 3.0, agar-agar 15.0, and trace element solution 1 mL/L). The mycelium was transferred to Eppendorf tubes, where a digestion buffer and small glass beads in the Precellys were used for DNA extraction. DNA was extracted under nitrogen at 3760 g for 3 cycles of 20 seconds each (20 second intervals) and then made ready for PCR. The amount of extracted DNA was determined using a NanoDrop photometer (Eppendorf BioSpectrometer). Then, PCR amplification of the rDNA region was performed in 50-µL reaction mixture using ITS1 (5’-TCCGTAGGTGAACCTGCGG-3’) and ITS4 (5’-TCCTCCGCTTATTGATATGC-3’) primers^29^. In the next step, 1 µL of template DNA (obtained from the isolate 329 at a concentration of 100–150 ng/µL) was added to the 24 µL master mix for PCR amplification of the rDNA region. The PCR program was as follows: Heat Lid at 110 °C; 1 cycle of 30 s at 98 °C; 35 cycles of 10 s at 98 °C, 30 s at 48.5 °C, 90 s at 72 °C; and a final cycle of 10 min at 72 °C (Bio-Rad). Following the PCR process, the size of amplified DNA was estimated to be approximately 600 bp on the agarose gel. The rDNA was extracted using the innuPREP DOUBLEpure Kit. DNA concentration was verified using the NanoDrop photometer (6 ng/µl is sufficient for 600 bp DNA). Finally, the samples were sent to Microsynth/Seqlab. The rDNA region sequence was used to perform a BLAST search within the GenBank database^30^.

### Cultivation conditions

A colony of *T. hirsuta* was produced on an SNL-Agar medium. Five mycelial discs (6 mm in diameter) were cut from the active growing edge of the colony. The mycelial discs were inoculated into 100 mL of potato malt peptone medium (PMP). Preculture in the medium was performed at 150 rpm for 4 days at 28 °C. The inoculant was prepared by homogenizing the cell suspension with a Waring laboratory blender (Heidolph Silent Crusher M, Germany) for 25 s at one-minute intervals before harvesting the pellets and washing three times with sterile distilled water (SDW). This mycelium suspension served as an inoculant for all experiments^31^.

For enzyme production, *T. hirsuta* isolate 329 was cultivated for 10 days at 125 rpm and 28 °C in complete darkness in Modified Kirk Broth containing (g per liter) (glucose 10, soytone 5, yeast extract 1, wheat bran 0.2, ammonium tartrate 2, CaCl_2_.H_2_O 0.1, MgSO_4_.7H_2_O 0.5, KH_2_PO_4_ 2, and trace element solution 1 mL. The pH was adjusted to 5.0). After the cultivation period, the fungal cultures were harvested and filtered (0.45 μm) to separate the mycelium. The resulting filtrate of *T. hirsuta* was used as an enzyme source for AFB_1_ biodegradation. This cultivation condition was selected based on previous optimization studies conducted for another organism (*Panus neostrigosus*) for AFB_1_ degradation, which provided a foundation for identifying effective parameters for enzyme production and activity^27^.

### Analysis of residual AFB1 by microplate reader

The entire reaction mixture, totalling 300 µL, was prepared for evaluation using a microplate reader. To initiate the process, 135 µL of culture supernatant derived from *T. hirsuta* was combined with 150 µL of a sodium acetate solution at pH 5.5. The 15 µL of AFB_1_ was added to the reaction mixture and the final volume was made up to 300 µL. The final concentration of the mixture is 5 µg/mL. The absorbance at 365 nm was recorded for one hour in a microplate reader (device and manufacturer) at 28 °C.

Two negative controls were used: one with culture supernatant containing autoclaved (inactive) enzymes and AFB_1_, and the other with active filtrate but without AFB_1_. The experiment was repeated three times with three parallel measurements each. This robust experimental design aimed to ensure the reliability and reproducibility of the results. The decrease in absorbance over the one-hour interval provided information regarding the degradation rate. The purpose of this experiment was to quickly check enzyme activity^27^.

### Analysis of residual AFB1 by HPLC

The AFB_1_ biodegradation rate of *Trametes hirsuta* was determined using a reaction mixture composed of 500 µL of sodium acetate buffer (0.1 M, pH 5.5), 485 µL of culture liquid, and 15 µL of AFB_1_ solution (final concentration: 1.5 µg/mL). Control reactions were conducted without the enzyme to account for non-enzymatic degradation. The reaction mixture was incubated in the dark at 28 °C for 1 h before being stopped with 500 µL of chloroform. After this process, the mixture was centrifuged at 239 g for 15 min, and the lower chloroform layer was evaporated to dryness at room temperature. This process was replicated in triplicate to ensure reproducibility and reliability of results. The AFB_1_-containing residue was re-dissolved in 200 µL of methanol by vortexing for 1 min to ensure complete dissolution. No distillation step was used for this process. The methanol extract was directly transferred into a clean HPLC vial for analysis^26^. The sample was analysed by HPLC (Shimadzu) under the following conditions: HP 1050 HPLC System Ti Series (Hewlett–Packard), C18 Column (150 mm x 4,6 mm, 5-µm particle size, 110 Å pore size; Gemini Phenomenex, Aschaffenburg, Germany); flow rate: 1 mL/min; UV detector with 365 nm. A standard curve was prepared using various AFB_1_ concentrations to calculate the biodegradation rates in all experimental groups.

### Protein purification

The culture liquid was harvested after 10 days of submerged fermentation, as optimization studies from previous research identified this as the optimal time for enzyme production. This liquid was then used as the source for protein purification using Fast Protein Liquid Chromatography (FPLC) and ultrafiltration. Partial purification was performed to determine the enzyme with the most effective AFB_1_ degradation capacity among those produced by *T. hirsuta*. Initially, the culture liquid of the isolate was centrifuged (at 11180 g and 4 °C), followed by filtration (using a 10,000 MWCO ultrafiltration tube at 4 °C and 2260 g). Then, protein fractions extracted from the culture liquid of the isolate were isolated by FPLC using a solution consisting of 50 mM bisTris (pH: 5.5) and 1 M NaCl in a 1:1 ratio. These protein fractions were visualised using sodium dodecyl sulphate-polyacrylamide gel electrophoresis (SDS-PAGE) with a 10% SDS concentration. A well was designated for the marker, and 10 µL of each sample was loaded into the remaining wells. Separated proteins were stained using Coomassie Brilliant Blue R-250 (Sigma-Aldrich), and their molecular masses were determined by comparison with low-range molecular mass markers from Merck (Darmstadt, Germany)^31^.

The next purification step involved ultrafiltration. To separate the two major proteins of FPLC fraction 6 this fraction was subjected to filtration by centrifugation at 3,368 rpm at 4 °C using 50,000 MWCO ultrafiltration tubes. Filtrate and retentate were analysed in the microplate reader assay (2.4) and SDS-PAGE with a 10% SDS concentration and staining with Coomassie Brilliant Blue R-250.

### Peptide mass fingerprinting

To identify the responsible enzyme, peptide mass fingerprinting was performed. The respective protein band was excised from a SDS gel, cut into small pieces, dried and incubated (30 min at 56 °C) with dithiothreitol (20 mM in 0.1 M NH_4_HCO_3_). After discarding the supernatant, the gel pieces were rehydrated in iodine acetamide solution (55 mM in 0.1 M NH_4_HCO_3_) for 30 min at ambient temperature in the dark. The supernatant was discarded, and proteins were digested using trypsin (34 U/mL, sequencing grade; Promega, Madison, WI, USA) in 0.1 M NH_4_HCO_3_ (37 °C for at least 4 h). The tryptic peptides were identified by nLC-qTOF-ESI-MS/MS.

Samples were injected into a nano-liquid chromatography system (EASY-nLC II, Bruker Daltronik, Bremen, Germany) equipped with a 20 mm pre-column (C18-A1 3PCS; ThermoFisher Scientific, Dreieich, Germany) and a capillary column (0.15 × 250 mm) packed with Grace MS C18 (3 μm particles, 300 Å pore; Grace Discovery Sciences (Columbia, SC, USA). Oligopeptides were eluted by a linear gradient (300 nL/min) of water and acetonitrile (each with 0.1% formic acid (v/v)) from 95% water to 95% acetonitrile within 25 min and held for 15 min. The nano-LC system was connected to a MaXis impact QTOF mass spectrometer (Bruker Daltronik) equipped with a captive nanospray ion source for electrospray ionization in the positive mode. The orthogonal time-of-flight mass analyser was calibrated before analysis (ESI-Low Concentration Tuning Mix, Agilent Technologies, Santa Clara, CA, USA) and operated with an average mass resolution > 30,000. Collision-induced ms/ms spectra (Ar) were recorded, and peptides from m/z 200 to 2000 were recorded and evaluated using the Protein Scape 3.0 software (Bruker Daltronic, Bremen, Germany). Oligopeptide sequences were identified using the Mascot search algorithm and the NCBI protein database (NCBInr, Taxonomy Fungi, 15.02.2023)^32^.

The enzymatic activity was calculated using the Michaelis-Menten equation: where v is the reaction velocity, Vmax is the maximum velocity (11.49 µM per minute), Km is the Michaelis-Menten constant (23.67 µM), and [S] is the substrate concentration.

**Calculation of *V***_***max***_:

$$v=\frac{{{\text{Vmax X }}\left[ {\text{S}} \right]}}{{{\text{Km}}+\left[ {\text{S}} \right]}}$$

### Statistical analysis

For statistical evaluation, each experiment was performed in triplicate, and the resulting data were expressed as mean values ± standard deviation (SD). Statistical significance of differences between experimental groups and controls was assessed using one-way ANOVA. For all analyses, statistical significance was defined as *p* <0.05.

## Results

### Biodegradation agent

**Fig. 2:**
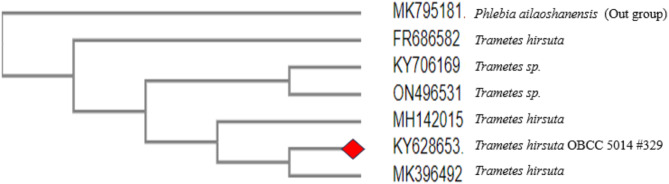
The phylogenetic tree based on ITS rDNA gene sequences of isolate 329 and representatives of the allied taxa.

The fruiting body sample of the isolate was determined to belong to the *Trametes hirsuta* species by traditional methods. Moreover, when the ITS rDNA region sequence of isolate 329 was analysed, it was found that it shared 99% genetic similarity with sequences found in the NCBI GenBank under accession numbers MK396492.1, FR686582.1, and KY706169.1. Figure 2 shows that this isolate falls within the evolutionary branch of *Trametes hirsuta*. As a result, it was concluded that isolate 329 was indeed a representative of the *Trametes hirsuta* species.

### Supernatant analysis with microplate reader

The quantitative assessment of the rate of AFB_1_ degradation hinged on the systematic measurement of absorbance decrease over the designated one-hour timeframe. This method provided a comprehensive understanding of how enzyme activity varied over time, contributing to a more nuanced interpretation of the degradation kinetics.

In establishing negative controls, autoclave-inactivated supernatant and active filtrate without AFB_1_ were chosen to serve as baseline references. The absorbance values derived from these negative controls set the standard against which the absorbance changes within the experimental group were evaluated. This meticulous control not only validated the specificity of the enzymatic degradation process but also allowed for a more precise determination of the actual impact of the active enzymes, ensuring that any changes observed were specifically related to AFB_1_ degradation. The inclusion of both controls further strengthens the reliability of the experimental results (Table 1).

**Table 1:** AFB_1_ degradation by Supernatant and FPLC fractions.

Active matter	Incubation time (h)	Absorbance decrease (365 nm)	AFB1 Degradation (%)^*^
Supernatant	1	X	X
Supernatant (inactivated)	1	0	0
Supernatant (w/o AFB1)	1	0	0
FPLC-F1	1	54.29	15.86
FPLC-F2	1	11.13	3.25
FPLC-F3	1	14.92	4.36
FPLC-F4	1	9.58	2.80
FPLC-F5	1	11.88	3.47
FPLC-F6	1	136.00	39.73

*Compared to the initial amount of AFB_1_ (F1-F6; FPLC Fractions 1 to 6).

Upon analysis, the rate of AFB_1_ degradation was quantified at 39.73% in one hour, providing a numerical representation of the efficiency of the enzymatic degradation process under the given experimental conditions. This quantitative metric not only reinforces the reliability of the experimental design, but also adds a critical dimension to our understanding of the enzyme’s effectiveness in degrading AFB_1_.

### HPLC confirmation of AFB1 biodegradation by pure enzyme

To assess the AFB_1_ degradation ability of the purified enzyme, a thorough evaluation was conducted through a 24-hour incubation period. Subsequent to this incubation, HPLC analysis was employed to quantify the extent of AFB_1_ degradation. The results revealed a substantial degradation rate, with a remarkable 77.9% reduction in AFB_1_ concentration.

### Fast protein liquid chromatography (FPLC) analysis

After the preliminary purification using a 10,000 MWCO ultrafiltration, further purification was conducted through FPLC, resulting in the separation of six fractions. Notably, the sixth fractions exhibited remarkable efficacy in degrading AFB_1_, as confirmed by microplate reader measurements. The three parallel measurements revealed an average AFB_1_ degradation rate of 57.28%, underscoring the potency of the enzymes within this specific fraction.

This observed degradation efficiency not only highlighted the success of the purification process but also emphasizes the importance of selectively isolating enzyme fractions for enhanced enzymatic activity. The identification of a fraction with such notable AFB_1_ degradation capability holds significant promise for advancing our understanding of *T. hirsuta*-produced enzymes and their potential applications in bioremediation efforts.

The purification of the enzyme capable of degrading AFB_1_ was initiated *via* FPLC. Following this chromatographic purification step, a basic assessment of protein composition was performed using Sodium Dodecyl Sulphate-Polyacrylamide Gel Electrophoresis (SDS-PAGE).

**Fig. 3:**
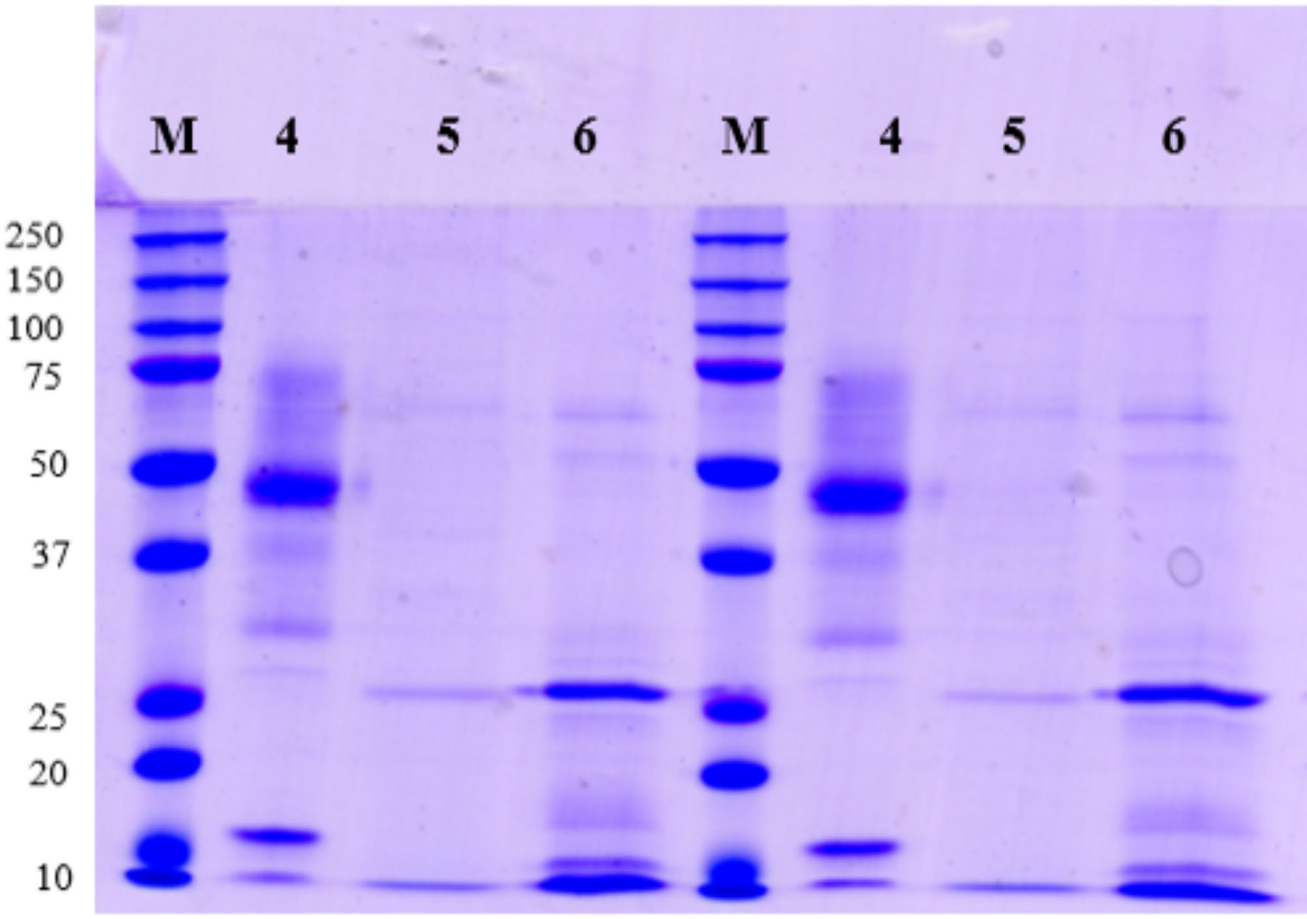
SDS-PAGE Gel of purified proteins. M: Marker. 4: Flask Number 4. 5: Flask Number 5. 6: Flask Number 6 (Flasks 4, 5, and 6 correspond to fractions collected during the FPLC purification process. Each flask contains proteins eluted at specific retention times representing different fractions based on their biochemical properties.)

The SDS-PAGE gel, upon electrophoresis followed by Coomassie Brilliant Blue staining, showed distinct protein bands. In particular, two distinct bands were observed in the purified sample, suggesting the presence of more than one protein. The molecular masses of these bands were estimated based on the migration of known molecular mass marker kits. One of the bands was determined to be 36 kDa, and the other was determined to be 55.8 kDa (Fig. 3).

### Ultrafiltration for band separation

**Fig. 4:**
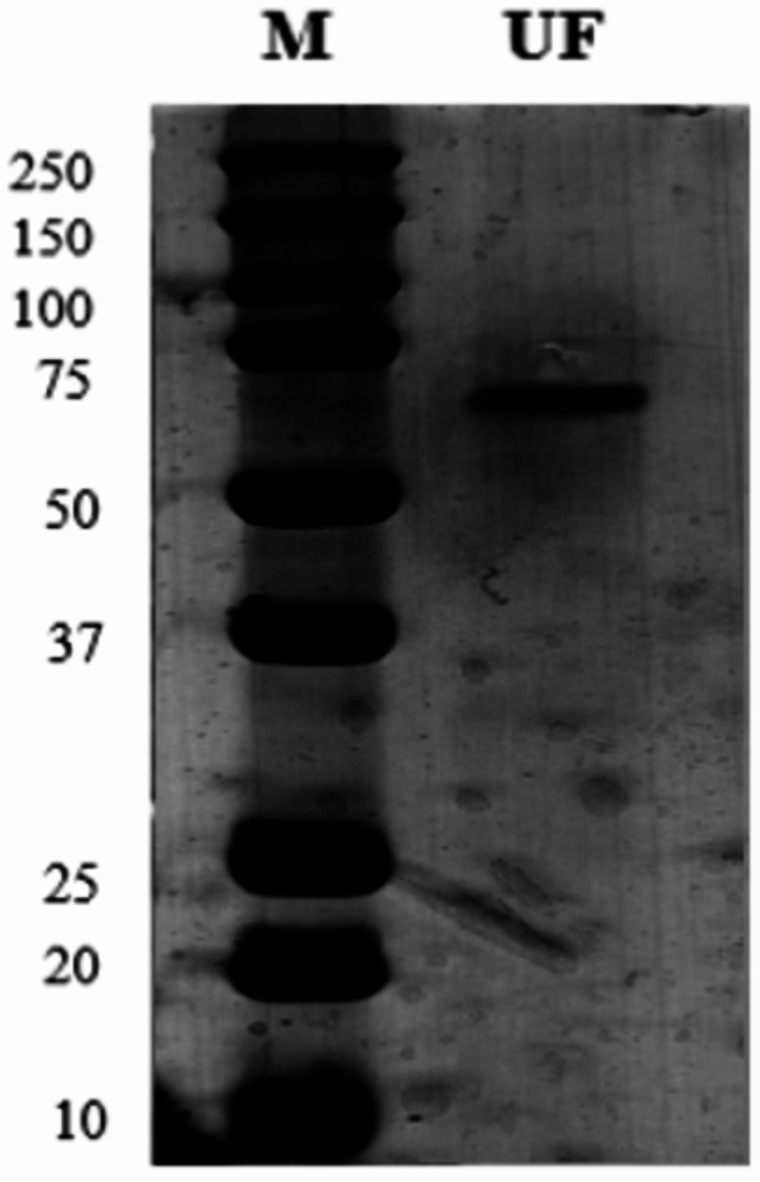
SDS-PAGE Gel; M: Marker. UF: 50.000 MWCO Ultrafiltration. “The original uncropped blot images for Fig. 4 are provided as Supplementary Information (Figure S1). The blot images were cropped for clarity, and full-length membranes with visible edges are provided for reference.”

As depicted in Fig. 3, two distinct bands were observed in the purified sample, corresponding to molecular weights of 36 kDa and 55.8 kDa, respectively. These bands were identified from Fraction 6, which was obtained from the FPLC purification process. Fraction 6 exhibited the highest AFB_1_ degradation activity during the preliminary purification experiments and was used for further analysis. Because one of the bands exhibited a mass exceeding 50 kDa, a subsequent purification process was conducted using a 50,000 MWCO ultrafiltration tube. The success of the separation was proven conducting SDS-PAGE of both filtrate and retentate. After this final step, we measured the AFB_1_ degradation for both bands. The results showed an AFB_1_ degradation rate of 77.9% for the 55 kDa enzyme and of the 36 kDa. After this final purification step, SDS-PAGE was performed again to confirm that the 55 kDa enzyme remained as a single band. In the final purification, the target enzyme was observed as a single band (Fig. 4).

### LC/QTOF/MS-MS analysis

**Fig. 5:**
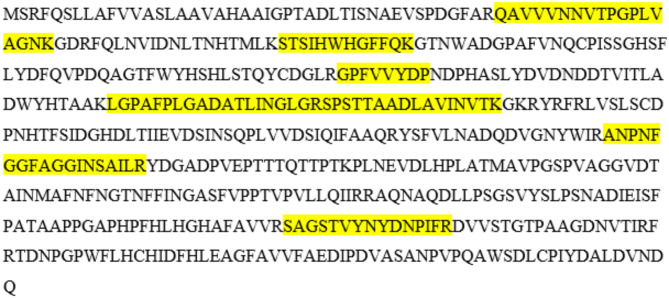
Identification of a lignolytic phenoloxidase [*Trametes hirsuta*]. Peptides found (single score > 40) are highlighted in yellow.

The protein band corresponding to the AFB_1_ degradation activity was excised from the SDS-PAGE gel and subjected to tryptic digestion, followed by peptide mass fingerprinting using nano-LC/QTOF/MS-MS. The Mascot database search (NCBI, taxonomy fungi) identified the protein as a lignolytic phenoloxidase from *Trametes hirsuta* (Accession Number: AAA33104.1) with a molecular weight of 55.848 kDa, a high score of 1643, and sequence coverage of 20.5% (Fig. 5).

### Enzyme kinetic calculation

**Fig. 6:**
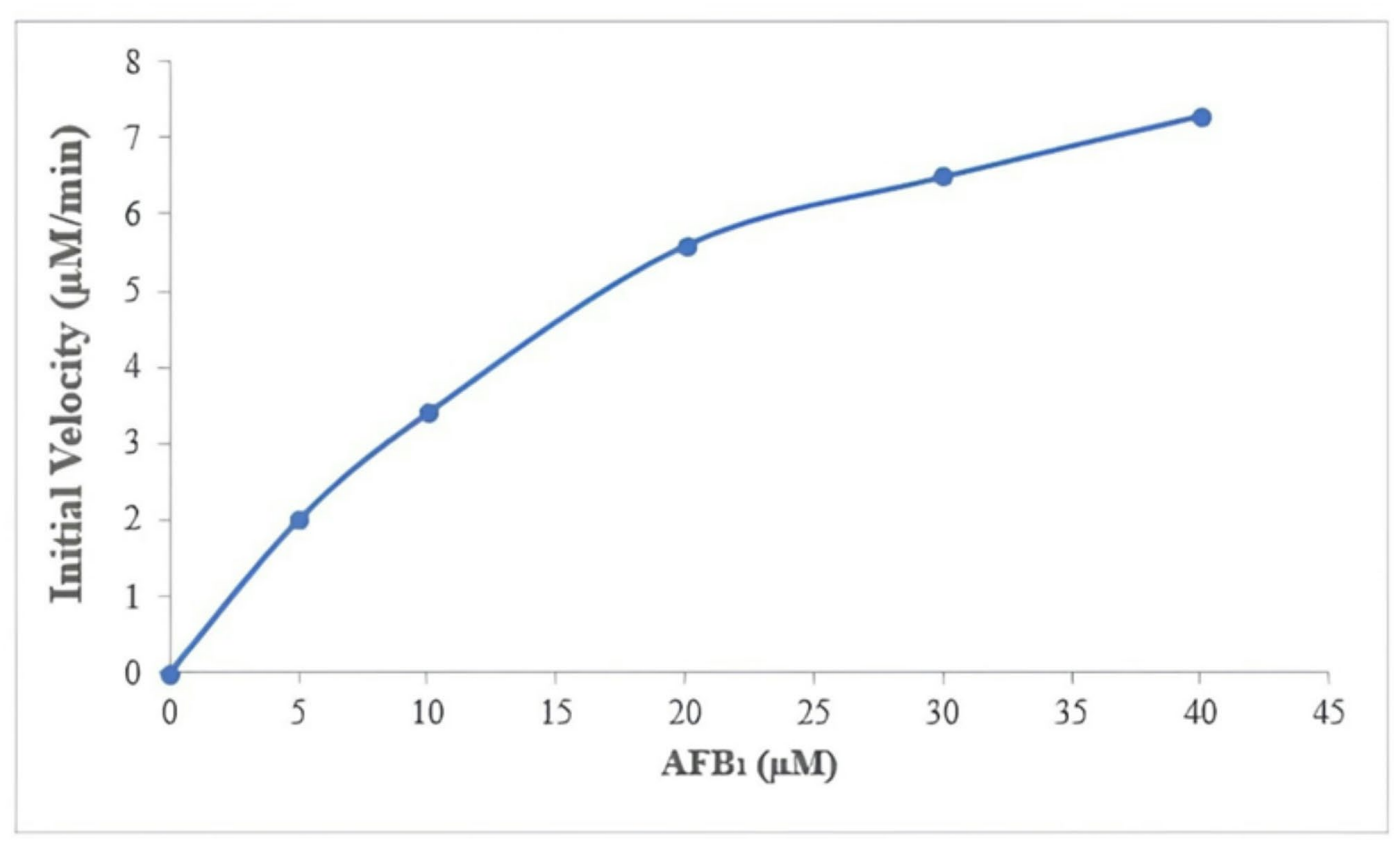
Enzyme kinetic curve.

The enzyme purified from *Trametes hirsuta* demonstrated substantial catalytic activity in degrading AFB_1_, with a maximum velocity (Vmax) of 11.49 µM per minute, reflecting its efficient conversion rate. This high Vmax suggests that the enzyme can process a significant amount of AFB_1_ per unit of time, highlighting its potent catalytic capacity. Furthermore, the Michaelis-Menten constant (Km) of 23.67 µM indicates a strong affinity of the enzyme for AFB_1_, which is characteristic of effective catalysis even at low substrate concentrations (Fig. 6).

## Discussion

This study delves into the biodegradation of AFB_1_, a potent mycotoxin with profound implications for human and animal health. The investigation focuses on *Trametes hirsuta* and its extracellular enzymes, seeking to identify and characterize the specific enzyme responsible for AFB_1_ degradation. The purification process, employing FPLC and ultrafiltration, led to the isolation of a single enzyme identified as a lignolytic phenoloxidase through tryptic peptide sequencing.

The characterised lignolytic phenoloxidase, with a molecular mass of 55.6 kDa, emerged as a key player in AFB_1_ degradation, demonstrating an impressive degradation rate of 77.9% under conditions. These findings significantly contribute to our understanding of the AFB_1_ biodegradation mechanism employed by *Trametes hirsuta*. The identification of the lignolytic phenoloxidase not only elucidates the biochemical basis of AFB_1_ degradation but also opens avenues for practical applications in mycotoxin remediation.

There is a risk of mycotoxin contamination in various agricultural products, including oilseeds, nuts, dried figs, grains, and more. Over the past decade, there have been 4752 mycotoxin notifications globally for food products on the Rapid Alert System for Food and Feed (RASFF). Notably, 63% (3000) of these notifications were related to ‘nuts, nut products, and seeds.’ A significant majority, 95% (2669), were attributed to Aflatoxins^32^. Efficient measures can be implemented on an industrial scale to prevent the proliferation of aflatoxins, which exhibit a fluorescent effect, in these products. To achieve this, a partially or fully purified enzyme can be applied to the surface of the nuts.

Some studies have previously identified enzymes effective in AFB_1_ degradation. For instance, laccase from *Phanerochaete chrysosporium* has demonstrated degradation potential with a rate of 13.8% over 72 h^24^. Similarly, manganese peroxidase from *Pleurotus ostreatus* achieved a remarkable degradation rate of 90% within 48 h^27^. These findings underscore the potential of fungal lignolytic enzymes in mycotoxin remediation. The enzyme achieved a degradation rate of 77.9% under straightforward conditions, without requiring hydrogen peroxide or other co-substrates. This characteristic enhances its practicality for real-world applications, such as treating the surfaces of contaminated fruits, vegetables, and nuts. Unlike catalases or peroxidases, which depend on hydrogen peroxide, lignolytic phenoloxidases only require oxygen, making them a more sustainable and efficient option for mycotoxin detoxification. In the present study, the lignolytic phenoloxidase from *Trametes hirsuta* exhibited a high degradation rate of 77.9%, highlighting its efficacy and potential application in industrial settings. Incubation periods for these enzymes ranged from one to 72 h, with observed degradation rates during these periods ranging from 15 to 100%. Table 2 provides a comparison of source organisms, enzymes, incubation times, and degradation rates from recently published work^33,34^.

**Table 2:** AFB_1_ degradation enzymes source and rates^33,34^.

Organism name	Enzyme	Incubation period (h)	Degradation rate (%)
*Armillariella tabescens*	AFO-ADTZ	NS	NS
*Phanerochaete chrysosporium*	Laccase	72	13,77
*Pleurotus ostreatus*	Manganese Peroxidase	48	90
*Bacillus subtilis*	Laccase	NS	98
*Irpex lacteus*	Manganese Peroxidase	9	100
*Panus neostrigosus*	NS	1	49
*Trametes versicolor*	AFB_1_-degrading enzyme	5	68
*Trametes hirsuta* (this study)	Lignolytic phenoloxidase	24	77

*NS not specified.

*P. ostreatus* and similar white rot fungi was recognised for producing various phenoloxidase derivatives^35^. The lignolytic phenoloxidase detected in this study, was previously identified as an extracellular enzyme produced by *T. hirsuta* with a 99.6% sequence identity to the enzyme from *T. hirsuta* described in^36^. *Trametes versicolor* produced an effective AFB_1_ degradation enzyme (TV-AFB_1_D), too^37^. Lignolytic enzymes, including laccase, manganese peroxidase, and lignin peroxidase, are widely distributed in higher fungi and have found first industrial applications. Of particular note, laccases play a pivotal role in the food industry, significantly improving the production, efficiency, and quality of various products such as beer, wine, fruit juice, and baked goods. Numerous studies indicate the positive impact of lignolytic enzymes when employed in food applications. For instance, laccases find applications in the beverage and food industry, manganese peroxidase is employed in the production of natural aromatic flavours, and lignin peroxidase catalyses the natural conversion of a reaction, resulting in the production of vanillin^38^.

In future investigations, the isolation of the gene and its expression in a heterologous host will be undertaken to confirm the gene’s identity, including its sequence and functional characteristics, as well as its putative action. Additionally, studying the reaction products of this enzyme reaction will provide guidance for research on aspects such as the toxicity of the AFB_1_ reaction products. Looking at the general working principle of phenoloxidases, it is supposed that higher-molecular condensed phenols will result, which do not bear the critical hepatological epoxy group formation of AFB_1_ anymore.

The lignolytic phenoloxidase identified in this study addresses critical limitations of existing AFB_1_ degradation approaches. Unlike other reported enzymes—such as laccases and manganese peroxidases, which necessitate complex reaction conditions—our enzyme requires only ambient oxygen, simplifying its application on the surfaces of fruits, vegetables, and nuts. Moreover, the rarity of lignolytic phenoloxidases in enzymatic detoxification studies, as evidenced by the sparse literature (one PubMed entry in the past decade), highlights the novelty of this work. This positions *Trametes hirsuta* and its enzyme as a cornerstone for advancing biological remediation strategies for mycotoxins.

Previous studies have explored various enzymatic strategies for AFB_1_ degradation. Yang et al. achieved up to 61% AFB_1_ removal in rice using the TVAFB_1_D gene from *Trametes versicolor*, although the specific enzyme type was not identified. Liu et al. reported that oxidoreductases from *Bacillus pumilus* were effective against AFM_1_; however, the degradation rates were not quantified. Brana et al. demonstrated over 50% AFB_1_ removal within one day and over 90% after seven days using manganese peroxidase and laccase enzymes derived from *Pleurotus eryngii* waste substrates. Schmidt et al. achieved complete AFB_1_ degradation in transgenic maize expressing a gene from *Armillaria tabescens*, though public concerns about genetically modified organisms (GMOs) have limited its applicability^39–42^.

Building on these efforts, the present study highlights the potential of lignolytic phenoloxidases from *Trametes hirsuta* as effective and sustainable tools for AFB_1_ biodegradation under mild conditions. These enzymes demonstrate promising applications in food safety and environmental protection. Nevertheless, further advancements are needed to improve enzyme stability and cost-effectiveness, ensuring the full potential of this approach is realized.

## Conclusion

This study underscores the potential of the lignolytic phenoloxidase from *Trametes hirsuta* as a powerful tool for addressing the critical issue of AFB_1_ contamination. With a degradation rate of 77.9% under mild conditions, the enzyme offers a promising and sustainable solution for mycotoxin remediation, particularly in food safety applications.

Unlike other enzymatic strategies requiring complex conditions, this phenoloxidase operates with ambient oxygen, making it ideal for industrial use on surfaces of agricultural products such as nuts, grains, and dried fruits. The enzyme’s ability to work efficiently without intricate reaction requirements positions it as a standout candidate for practical applications.

Future research should focus on isolating the corresponding gene, expressing it in heterologous hosts, and analyzing the toxicity of reaction products to fully harness the enzyme’s potential. These advancements will not only confirm its identity and mechanism but also provide insights into its safety and efficacy in broader contexts.

In summary, this study establishes *Trametes hirsuta* and its lignolytic phenoloxidase as a cornerstone for advancing mycotoxin detoxification strategies, with potential benefits extending to environmental protection and industrial applications in the food industry. Its ability to produce extracellular lignolytic enzymes with stable activity under simple conditions makes it an ideal candidate for further investigation. By addressing current limitations and simplifying application processes, the findings pave the way for scalable, effective, and eco-friendly solutions to combat AFB_1_ contamination.

## Electronic supplementary material

Below is the link to the electronic supplementary material.

Supplementary Material 1

## Data Availability

The data used to support the findings of this study are included within the article. The ITS rDNA sequence generated during this study is available in the NCBI GenBank under accession numbers MK396492.1, FR686582.1, and KY706169.1. Protein sequence data identified in this study is associated with accession number AAA33104.1 in the NCBI database.
