# Supplementary material for: Aflatoxin B1 (AFB1) biodegradation by a lignolytic phenoloxidase of Trametes hirsuta
Source: Sci Rep. 2025 Feb 21;15:6330. doi: 10.1038/s41598-025-90711-y (PMC11845786; doi:10.1038/s41598-025-90711-y)
Supplement: Supplementary file 1 — Supplementary Material 1 [file 41598_2025_90711_MOESM1_ESM.docx]

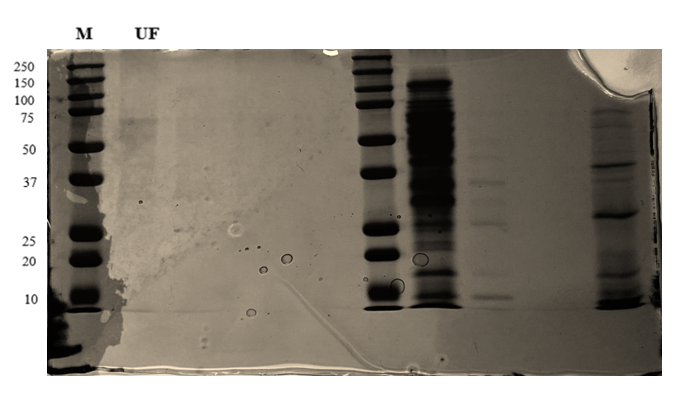


Original uncropped blot images corresponding to Figure 4 in the main manuscript. The images include full-length membranes with visible edges for transparency and compliance with the journal's editorial policies. Among the replicates performed, only the most representative gels with clear band patterns were photographed and included in the study. Other replicates were visually inspected and confirmed to show consistent results but were not photographed. This approach was taken to ensure clarity and focus on the most illustrative data. The absence of photographs for the remaining gels does not impact the reproducibility or reliability of the findings, as all experiments were repeated at least 3 times with consistent outcomes.
